# Impact of polygeNic risk score for glaucoma on psycHosocial ouTcomes (INSiGHT) study protocol

**DOI:** 10.1371/journal.pone.0312390

**Published:** 2024-12-26

**Authors:** Giorgina Maxwell, Robert Allen, Simone Kelley, Lucinda Hodge, Georgina L. Hollitt, Mathias Seviiri, Daniel Thomson, Joshua Schmidt, Jamie E. Craig, Sarah Cohen-Woods, Emmanuelle Souzeau

**Affiliations:** 1 Department of Ophthalmology, Flinders Medical and Health Research Institute, Flinders University, Adelaide, SA, Australia; 2 Department of Psychology, Flinders Medical and Health Research Institute, Flinders University, Adelaide, SA, Australia; 3 QIMR Berghofer Medical Research Institute, Brisbane, QLD, Australia; Public Library of Science, UNITED STATES OF AMERICA

## Abstract

Glaucoma is the leading cause of irreversible blindness with early detection and intervention critical to slowing disease progression. However, half of those affected are undiagnosed. This is largely due to the early stages of disease being asymptomatic; current population-based screening measures being unsupported; and a lack of current efficient prediction models. Research investigating polygenic risk scores (PRS) for glaucoma have shown predictive ability to identify individuals at higher risk. Potential clinical applications include identification of high-risk individuals, resulting in earlier diagnosis and treatment to prevent glaucoma blindness, and adjusted monitoring for low-risk individuals. However, the psychological impact of receiving glaucoma PRS is unknown. There is a critical need to evaluate risk information communication and assess the impact of receiving results, to support clinical implementation of glaucoma PRS testing. In this prospective study, 300 individuals from the GRADE (Genetic Risk Assessment of Degenerative Eye disease) study will be recruited to investigate the psychosocial impact of disclosing polygenic risk results for glaucoma. GRADE aimed to apply PRS testing on 1,000 unexamined individuals aged 50 years or older from the general population and examine a subset of these individuals to assess the clinical validity of PRS to detect glaucoma. In this study, individuals each from the bottom decile (10%), top decile (10%), and middle (45–55%) of the PRS distributions will be invited to receive research glaucoma PRS results. Participants who choose to receive their results will complete up to four questionnaires (prior to receiving their results, and subsequently two-weeks, six- and 12-months after receiving their result). The questionnaires will include health belief model measures and assess glaucoma anxiety, general anxiety and depression, test-related distress, decisional regret, and recall and understanding of results. This research will provide guidance for the implementation of polygenic risk testing into clinical practice and inform delivery strategies.

## Introduction

Glaucoma is the leading cause of irreversible blindness worldwide with at least half of those affected undiagnosed [1–4]. The condition is typically asymptomatic in the early stages; however, it can progress to loss of peripheral vision and total blindness if left untreated. There is strong evidence that interventions can slow disease progression and prevent associated visual loss, especially when patients are identified early in the disease process [5, 6]. Therefore, it is critical to identify high-risk individuals in early stages of the disease to reduce the extent of glaucoma-related blindness.

Population-based screening for glaucoma is currently not supported because it is not sufficiently cost-effective, sensitive, or specific [7–9]. Traditional risk factors such as age, ethnicity, and family history, which currently inform targeted screening, have limited risk stratification predictability [9, 10]. Additionally, it is currently difficult to predict which patients showing early signs of glaucoma will progress to developing the condition and associated vision loss. As a result, the lack of prediction models and insufficient screening guidelines can result in both delayed and over treatment [11].

Glaucoma is one of the most heritable of all common human diseases with heritability estimated at 70% [12]. It is a complex disease with a genetic architecture that includes both rare highly penetrant genetic variants (accounting for <5% of cases) [13] and common variants of smaller effect size. Individual common risk variants each have very small effect sizes and so individually have a minimal effect in predicting one’s risk of developing glaucoma. However, risk variants can be combined together and weighted by their magnitude of effect in the form of a polygenic risk score (PRS). Glaucoma PRS have shown predictive ability to identify individuals with glaucoma, improved risk prediction models when combined with traditional risk factors [10, 14–16], and currently explain 14% of familial risk [17].

Considering the irreversible nature of vision loss from glaucoma, the lack of effective screening measures, and the asymptomatic but treatable nature of the disease, the predictive ability of PRS shows potential clinical utility. Clinical applications for a glaucoma PRS could include improved screening models with earlier diagnosis and treatment for those at higher risk and reduced/adjusted monitoring for those at lower risk [18]. Polygenic risk testing for glaucoma is available commercially but is currently not part of standard practice. Previous research from our group showed acceptability, with 70% of individuals indicated that they would take the test if it was available [19]. To move toward clinical implementation of PRS testing, we need to evaluate how to effectively communicate information about risk and assess the potential impact of receiving results.

There are significant challenges associated with communicating the uncertainties inherent to the probabilistic nature of the test which may lead to potential harms [20, 21]. A recent systematic review on PRS communication for complex conditions reported no evidence of generalised or disease-specific anxiety, depression or negative psychosocial impact, and overall low levels of decisional regret [22]. Although studies identified no major long-term adverse effects of communicating PRS or risk estimates for different complex diseases, genetic testing-specific distress has been experienced by those in higher PRS risk groups [23, 24]. There is a current gap in our understanding of the psychosocial implications and long-term comprehension of PRS results for glaucoma. To the best of our knowledge, this study is the first to assess psychosocial outcomes of PRS communication for glaucoma.

## Methods

### Study framework

The health belief model (HBM) is the theoretical framework guiding this research. The HBM posits that an individual’s perception and beliefs about a health condition, and themselves, will determine if and how they adopt health behaviours [25]. These health beliefs are the perceived severity and susceptibility of the condition, perceived self-efficacy, benefits, and barriers of engaging in the health behaviour, and the cues to action to engage in the target health behaviour [26]. Health beliefs have been associated with psychological distress (depression and anxiety symptoms), whereby they directly correlate with distress or mediate the relationship between a health behaviour and distress [27, 28], including in the context of genomic testing [29].

### Study aims

This study aims to explore:

1. The comprehension and recall of PRS results
2. The psychosocial factors that predict response to PRS results
3. How PRS predictions may moderate or mediate psychological predictors
4. Whether HBM variables moderate or mediate psychological predictors

### Study design

**Fig 1:**
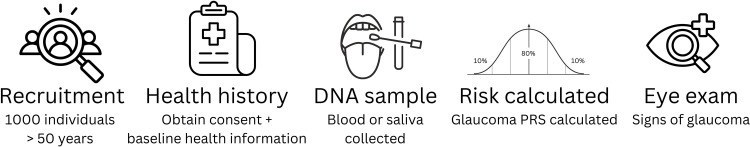
Summary of the GRADE study.

This prospective cohort study has been approved by the Southern Adelaide Clinical Human Research Ethics Committee (SAC HREC, ethics approval number: 2023/HRE0085) and adheres to the revised declaration of Helsinki. The project will be conducted by the Department of Ophthalmology at Flinders University. The study will leverage participants who are part of the ongoing GRADE (Genetic Risk Assessment of Degenerative Eye Disease) Study [30]. GRADE is a prospective study that aims at applying PRS testing previously developed in a minimum of 1,000 individuals aged 50 years and older from the general population, of any ethnicity, and clinically examine individuals from across the PRS risk spectrum to detect undiagnosed glaucoma or age-related macular degeneration (Fig 1). The GRADE exclusion criteria excludes from the study those aged under 50 years, or an inability to provide written informed consent. Individuals already diagnosed with glaucoma are neither excluded nor targeted. GRADE participants have been recruited through several approaches including eligible individuals who had previously participated in a questionnaire-based study assessing attitudes towards PRS testing for glaucoma, advertisement in public and private outpatient clinics, sporting venues and community clubs and organisations, and presentations about eye disease to community organisations.

Individuals recruited into this study will be invited to receive their research glaucoma PRS results from the GRADE study. They will receive a personalised patient-centred report based on their PRS results (low, middle, or high) previously developed by G.L.H, which was assessed by participants and revised based on their feedback [31]. The risk of developing glaucoma for the low (bottom decile) and high (top decile) risk groups was calculated by M.S for this study. The report includes participants’ absolute and relative risk compared to the general population (Pages 1–3 of S1 File for low, moderate and high risk), explains PRS, the meaning and limitations of results, glaucoma signs, risk factors, screening, treatment options, and resources (Page 4 of S1 File). Because no clinical guidelines currently exist for monitoring glaucoma based on PRS results, the reports include the existing Australian recommendations for the population to have regular eye health checks from the age of 50 years [7].

### Data management plan

Clinical and genetic data from the GRADE study will be accessed for this study. Participants will be informed of this verbally and in writing through the participant information sheet and consent form. Data sheets and consent forms will be stored as hard copies in lockable filing cabinets in the Department of Ophthalmology at Flinders University. All digital data will be stored on a secure network (SA Health) with de-identified data stored on the Flinders University network. Participant identity will only be known by the study investigators and members of the research team. Only members of the research team will have access to the data collected through this study. All participant information will be de-identified before data analysis and publication of study results in peer-reviewed journals.

### Participants

Approximately 300 individuals from the GRADE study will be invited to participate. The total sample size will include approximately 100 individuals each from the bottom decile (10%), top decile (10%), and from the middle (45–55%) of the PRS distributions. The sample size for this study will be constrained by the size of the GRADE cohort and the number of participants falling into each group. Recruitment started on 7^th^ July 2023 and is planning to be completed by 1^st^ December 2024.

Individuals who will not have the ability to understand and meet the requirements of the study will be excluded. Individuals with impaired decision-making capacity have not been enrolled in the GRADE study. Additionally, individuals who are not sufficiently proficient in English to be able to provide written informed consent and complete questionnaires in English will not be invited to the study.

### Recruitment process

**Fig 2:**
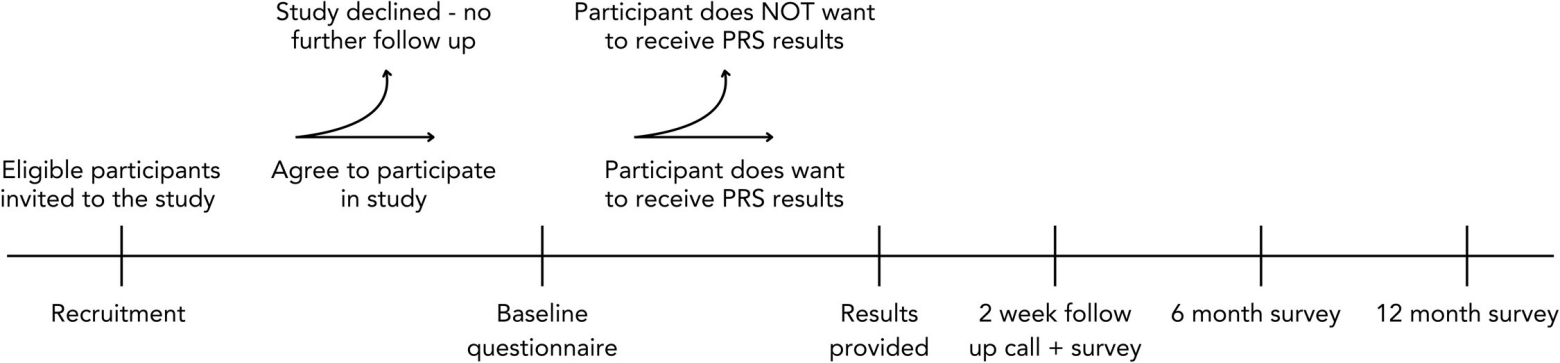
Study design and flow of participants through study.

The study design is detailed in Fig 2. Eligible participants will receive an invitation letter and a participant information sheet by email or post to participate, depending on their available contact details. They will be able to indicate their interest in participating and being contacted by returning a reply form enclosed with the invitation letter or replying by email. A member of the research team (R.A., L.H., S.K.) will follow up with participants by contacting them up to three times before follow up is ceased.

The implications of receiving genetic results, including potential psychosocial harms and impact on life insurance in Australia, will be discussed with participants at the time of enrolment and consent, prior to them deciding whether to receive their glaucoma PRS results. All participants will provide written consent. Participants who choose to receive their results (termed Receivers) will be invited to complete four questionnaires in total: at baseline (prior to receiving their results), two weeks, six, and 12 months after receiving their PRS result. Questionnaires will be provided by email or by mail depending on participants’ preferences. After completing the baseline questionnaire, they will be given the option to receive their PRS results by email or post. Participants will receive a follow-up call from a member of the study team within two weeks following receipt of their results. Participants who choose not to receive their result will be invited to complete a questionnaire at baseline only (termed Decliners). Participants will receive three reminders (by email or by phone) to complete the questionnaires sent. Data will be collected through self-reported forced-response questionnaires using REDCap (taking 15–30 minutes each to complete) designed to obtain repeated measures. Questionnaires completed by mail will be checked for completeness and entered manually into REDCap, and participants will be recontacted if data is missing from questionnaires returned by mail (R.A, L.H, S.K.). Receivers will receive two gift vouchers, one each after completing the 2-week and the 12-month questionnaires. Decliners will receive one voucher after completing the baseline questionnaire.

### Measures

The surveys have been designed to investigate the psychological impact (general anxiety, depression, stress, test-related distress), the different constructs of the HBM, genetic determinism, their attitude toward uncertainty, comprehension and recall of results, decisional regret, results expectation, and eye screening behaviour. A summary of which measures included at each time point is presented in Table 1 with detailed questionnaires presented in S2 File.

**Table 1:** Measures and questionnaires selected for study and timeline.

Measure	Receivers				Decliners
	Baseline	2w	6m	12m	Baseline
1. Sociodemographic characteristics	√				√
2. Eye screening behaviour	√				√
3. Glaucoma status	√		√	√	√
4. Health belief model measures					
a. Perceived glaucoma severity	√	√		√	√
b. Perceived glaucoma risk	√	√		√	√
c. Response efficacy (genetic results)	√	√		√	
d. Response efficacy (eye test)	√	√		√	√
e. Response cost (genetic results)					√
f. Response cost (eye test)	√	√		√	√
g. Self-efficacy (genetic results)	√	√		√	
h. Self-efficacy (eye test)	√	√		√	√
i. Cues to action	√	√		√	√
5. Uncertainty avoidance	√				√
6. Glaucoma anxiety	√	√	√	√	√
7. Knowledge of glaucoma	√	√			√
8. Genetic determinism	√	√		√	√
9. Stressful life events	√			√	√
10. Generalised anxiety, stress, & depression	√	√	√	√	√
11. Test-related distress		√	√	√	
12. Recall of results		√		√	
13. Results expectation		√		√	
14. Decisional regret		√	√	√	
15. Eye screening follow up				√

1. *Sociodemographic characteristics*: age, gender, family cultural background, ethnicity, level of education, employment status, language spoken at home, whether they have children.
2. *Eye screening behaviour*: last eye examination, frequency, and reason for eye examination, intention to have an eye examination the next 12 months.
3. *Glaucoma status*: whether they have personally been diagnosed with glaucoma, or high intraocular pressure. Whether anyone in their family has been diagnosed with glaucoma and if so how many and their relation to the participant.
4. Health belief model measures: These questions will use a six-point Likert scale to omit the midpoint to eliminate the possibility of participants misusing it [32] and adapted to be relevant to glaucoma.
  - *Perceived severity of glaucoma*: one question adapted from a previous study on inherited breast cancer [33].
  - *Perceived glaucoma risk*: three items adapted from a previous study [34]. Participants will be asked to rate their chances of developing glaucoma in the future from 0–100% as well as compared to individuals with same age and gender and similar family history of glaucoma on a five-point Likert scale.
  - *Response efficacy (genetic results)*: seven items to assess the perceived benefits of receiving genetic results, including four adapted from a previous study [34] and three we have previously developed for glaucoma [19].
  - *Response efficacy (eye test)*: seven items to investigate the perceived benefits of an eye test, with questions adapted from a previous study [34], based on reviewing published literature, and on researchers’ expertise.
  - *Response cost (genetic results)*: 12 items to assess the perceived barriers to receiving genetic results, including four adapted from a previous study [34] four we have previously developed for glaucoma [19], and four new items created based on reviewing published literature and researchers’ expertise.
  - *Response cost/barriers (eye test)*: nine items to evaluate the perceived barriers to having an eye examination adapted from two previous studies on glaucoma screening barriers [35, 36].
  - *Self-efficacy (genetic results)*: seven items to assess confidence in undertaking PRS testing in the context of barriers adapted from previous studies [33].
  - *Self-efficacy (eye test)*: 11 items to assess confidence in undergoing an eye examination adapted from previous studies on barriers to glaucoma screening [35] and glaucoma medication self-efficacy [37].
  - *Cues to action*: five items to assess ways that may prompt individuals to have an eye test adapted from a previous study on the health belief model scale for exercise [38].
5. *Uncertainty avoidance*: the Attitudes Towards Uncertainty scale was adapted to be specific to glaucoma [39]. It includes eight items scored on a five-point Likert scale where higher scores indicate a more negative attitude towards uncertainty.
6. *Glaucoma anxiety*: measured using the Impact of Events Scale (IES), a validated measure of frequency of intrusion, hyperarousal, and avoidance in the context of a specific event [40]. The IES comprises 15 items measured on a four-point Likert scale which was adapted to be specific to glaucoma.
7. *Knowledge of glaucoma*: 12 true/false items, with nine items adapted from a previous study [33] to be specific to glaucoma, and three questions developed based on existing literature and researchers’ expertise. These items were designed to assess participants’ knowledge of glaucoma, risk and genetic risk.
8. *Genetic determinism*: four items on six-point Likert scale to assess participants’ perceptions regarding to what degree glaucoma is determined by genetic and environmental risk factors, developed based on literature review and researchers’ expertise.
9. *Stressful life events*: measured using the List of Threatening Experiences scale previously developed [41] comprising 13 Yes/No questions.
10. *Generalised anxiety*, *stress*, *and depression*: assessed using the short 21 items Depression Anxiety and Stress Scale (DASS) [42]. The scale comprises seven items each to measure concepts of anxiety, stress, and depression experienced over the past week. They are scored on a four-point Likert scale with higher scores on the three subscales indicating higher levels of stress, anxiety, and/or depression.
11. *Test-related distress*: assessed using the Feelings About genomiC Testing Results (FACToR) questionnaire [43], validated in a cancer setting and derived from the Multidimensional Impact of Risk Assessment Scale (MICRA) for cancer-related distress [44]. This scale includes four subscales including negative emotions (three items), positive experience (four items), uncertainty (three items), and privacy concerns (two items), which are scored on a five-point Likert scale. Higher scores indicate greater psychosocial impact across subscales, except for positive experience where higher scores indicate lower impact.
12. *Recall and interpretation of results*: two items were developed to assess participants’ recall of their PRS results, one as an open question, and one asking them if their risk was higher, lower or the same as the general population to illustrate the results received by the three risk groups.
13. *Results expectation*: two new items were developed to assess this concept, specifically whether their results fit their expectations based on their personal and family history of glaucoma based on reviewing published literature, and on researchers’ expertise.
14. *Decisional regret*: assessed using the five items from the Decisional Regret Scale measuring participants’ decision satisfaction and conflict on a five-point Likert scale [45].
15. *Eye screening follow up*: four items were developed based on literature review and researcher expertise to assess actual eye screening behaviours since participants had received their PRS results, the reasons behind this, and whether they were aware of any progression or changes to their diagnosis since having received their results.

### Planned statistical analyses

Analysis will be performed using IBM SPSS Statistics (version 29). For association analysis linear and logistic regression will be used as appropriate for the outcome variables. Multivariable analyses will be conducted to adjust for potential confounding variables. As this is a novel study, demographic characteristics including age, sex, biological children, level of education and glaucoma diagnosis will be considered as potential confounders. Moderated regression will be conducted using the PROCESS (version 4.3.1) macro for SPSS [46]. Appropriate regressions will be used to investigate whether outcomes differ between the different PRS risk groups. Repeated measurements (two week, six months and 12 months post results) will be analysed using linear mixed models to assess whether outcomes change over time among Receivers.

## Discussion

Polygenic risk has the potential to dramatically change service provision and offer personalised genetic testing to a wider group of individuals. A high PRS score has been associated with earlier age at glaucoma diagnosis [10, 16, 47], more advanced disease [10, 15], progression [48] and treatment escalation [49]. Through individualised glaucoma PRS, clinicians may be able to determine which patients at higher risk would benefit from earlier diagnosis and interventions and alleviate the burden of unnecessary investigations and treatments for those at lower risk. Improved detection and diagnosis would minimise the personal and economic costs of glaucoma-associated vision loss as well as reducing workload resulting from over investigation and/or treatment.

While glaucoma PRS appears to be a promising approach for risk stratification and adjusting patient management, the implications of communicating these results have not been explored in the context of glaucoma. To the best of our knowledge, this study is the first to investigate the psychosocial impact and understanding of receiving PRS results for glaucoma. Understanding these effects is crucial to developing strategies to ensure patients accurately comprehend their risk and identify support needed.

A strength of this study is the exploration of the Health Belief Model (HBM) as a theoretical framework to evaluate the impact of PRS results on health behaviour and beliefs concerning glaucoma. The HBM is one of the most widely studied and applied models in understanding health behaviours across diseases [25]. The associated beliefs and behaviours have been demonstrated to affect psychosocial outcomes for common conditions [27–29]. Moreover, HBM-based interventions are effective in improving positive behavioural health outcomes [50, 51]. However, to date the role of health beliefs in the context of receiving genetic results has not been explored for glaucoma. By applying this model, our study aims to identify how health beliefs surrounding PRS might influence psychological distress and could guide the development of future interventions.

The design of the protocol include potential confounding factors to be taken into consideration. The manner in which PRS information is conveyed to participants plays a significant role in psychological responses to the genetic information. Communication of risk is known to influence risk perception, understanding and behavioural outcomes [52–55]. However, there is limited research on best practice methods for reporting and communicating PRS [56, 57]. An additional strength of the study is the use of a patient-centred PRS report tailored to the participants’ polygenic risk group (low, middle or high). The PRS reports have been previously developed by our group based on a review of the existing literature and informed by participants’ feedback and preferences with content and format, including preferred representation of risk [31]. They incorporate written numerical and graphical display of risk, provide absolute risk contextualised to population risk, simple information and resources. The user-centred approach to the design was applied to facilitate communication and understanding across different levels of numeracy and health literacy.

Recommendations associated to different results and risk groups may influence psychosocial outcomes. Previous research has emphasised the importance of standardised clinical guidelines for PRS integration into clinical practice [20, 58]. This is supported by a recent practice resource from the National Society of Genetic Counsellors [57], and a position statement from the Human Genetics Society of Australia on the clinical implementation of PRS [56]. However, there are currently no clinical guidelines to determine how PRS should guide an individual’s screening and/or management for glaucoma. Consequently, participants in this study are provided with the screening recommendations for the general population and recommendations are not tailored to the genetic risk. The study will assess whether the lack of personalised recommendations based on genetic risk might impact behavioural outcomes.

For some time now, participants and healthcare professionals have expressed concerns over the potential for genetic discrimination by insurance companies [59]. Some countries (such as Australia, USA, Canada, UK) have implemented laws or moratoria to prevent the use of genetic results for health insurance products [60–63]. However, the potential impact of PRS on insurance products, including health and life insurance is not currently known. The implications regarding the potential use of PRS in personal insurance in the context of the Australian moratorium are discussed with participants at enrolment in this study. Potential genetic discrimination may deter individuals from participating and learning their glaucoma PRS and lead to recruitment bias. Therefore, participants who decline to receive their PRS results are offered to complete a questionnaire to assess potential factors.

This study also has some limitations. It is beyond the capacity of the research to translate the patient questionnaires into other languages. As such, individuals who do not have a sufficient level of English to be able to provide written consent in English and participate in the questionnaires are not recruited. Recruitment is restricted by the total number of participants enrolled in the larger GRADE study (n = 1,000) and participants who consent to receive the PRS results. There are currently no validated measures assessing the health belief model measures (perceived benefits, perceived barriers, self-efficacy), glaucoma anxiety or genetic-related distress for glaucoma. Measures were therefore selected that had been validated for other complex diseases such as familial cancer for their similarities with the perceived severity, actionability, and genetic architecture to glaucoma. As such, further studies will be needed to replicate results.

In conclusion, this study aims to explore the psychosocial and behavioural outcomes of providing personalised glaucoma PRS scores for individuals from the general population. Outcomes will inform appropriate delivery strategies and support to patients and healthcare professionals for implementation of this innovative service model into clinical practice.

## Supporting information

S1 File Polygenic risk scores reports. The reports show the low risk (page 1), middle risk (page 2) high risk (page 3) and supporting information common to all three risk groups (page 4).
(PDF)

S2 File Questionnaires. (DOCX)
