# Supplementary material for: Impact of polygeNic risk score for glaucoma on psycHosocial ouTcomes (INSiGHT) study protocol
Source: PLoS One. 2024 Dec 26;19(12):e0312390. doi: 10.1371/journal.pone.0312390 (PMC11670974; doi:10.1371/journal.pone.0312390)
Supplement: S1 File — The reports show the low risk (page 1), middle risk (page 2) high risk (page 3) and supporting information common to all three risk groups (page 4). (PDF) [file pone.0312390.s001.pdf]

Polygenic Risk Score (PRS) Report - Glaucoma

Flinders University

Firstname: Surname: ID:

DOB: Date of reporting:

1. **PRS Result - Glaucoma**

Your result - low risk

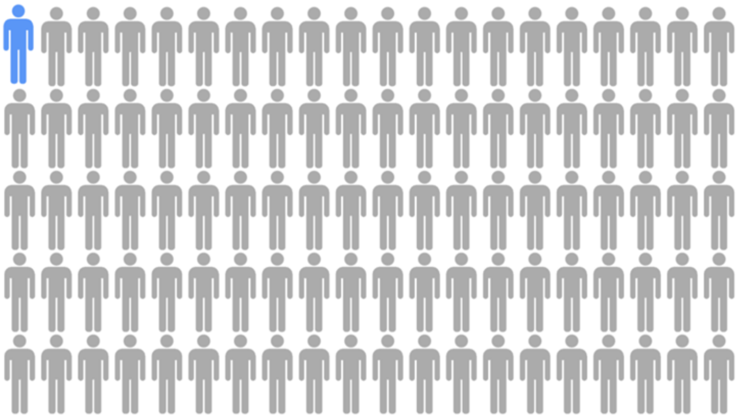

Your lifetime genetic risk of developing glaucoma is 1%. This means that 1 out of 100 people with the same polygenic risk result as you will develop glaucoma.

Average population

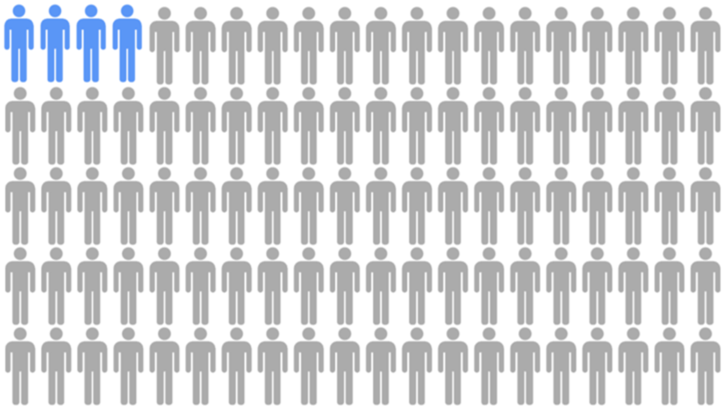

The average population lifetime genetic risk of developing glaucoma is 4%. This means that 4 out of 100 people with an average polygenic risk will develop glaucoma.

2. **What does my test result mean to me?**

- Your result suggests you are 4 times less likely to develop glaucoma compared to other people with an average genetic risk.
- Your result does not mean that you have glaucoma now.
- Even if your result shows that you are at low risk, you could still develop glaucoma.
- This result does not change your current screening or management program. This means that you should continue to follow advice from your eye care provider if you have one.
- The current recommendations for the population are to have regular eye health checks from the age of 50 years.
- Please refer to the back of the page for more information on glaucoma.

3. **What are the limitations of the test?**

- <u>These results have not been validated in an accredited testing laboratory. Therefore, they should not be used as part of your clinical management.</u>
- PRS results represent a probability of individual disease risk and are therefore not diagnostic.
- This result may change with time as new versions of the PRS become available.
- This test only estimates your risk of primary-open angle glaucoma (the most common subtype of glaucoma). It does not estimate your risk of other types of glaucoma or other conditions.
- Although the polygenic score predicts risk in many ancestries, it has been best validated in individuals of European ancestry.
- This test does not account for all genetic changes known to cause glaucoma. Therefore, your risk may be higher, especially if you have a strong personal family history of glaucoma.
- Results should be interpreted in conjunction with other known clinical risk factors. It does not take into account non-genetic risk factors.

Polygenic Risk Score (PRS) Report - Glaucoma

Firstname: Surname: ID:

DOB: Date of reporting:

1. PRS Result - Glaucoma

Your result - average risk

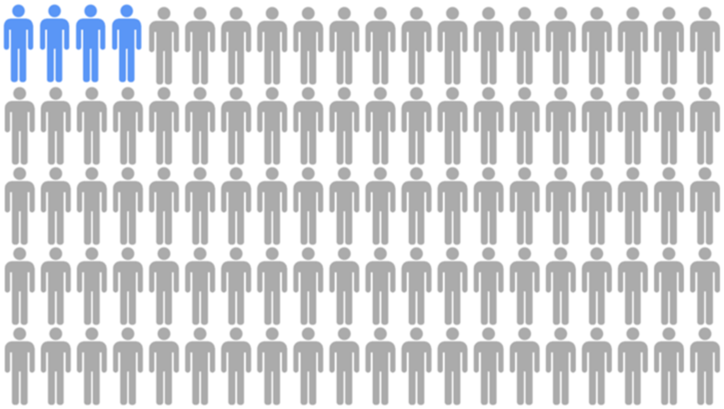

Your lifetime genetic risk of developing glaucoma is 4%. This means that 4 out of 100 people with the same polygenic risk result as you will develop glaucoma.

Average population

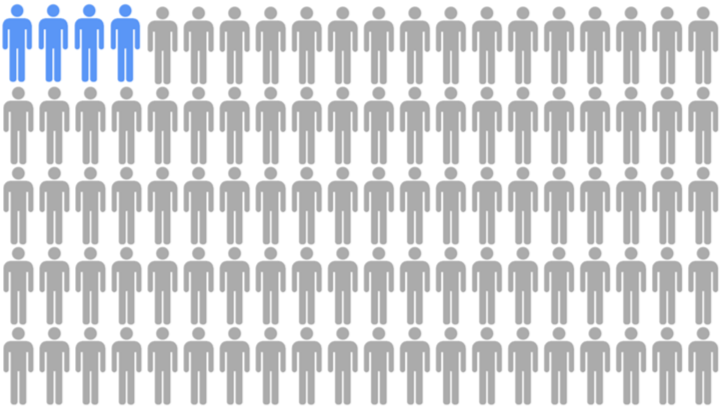

The average population lifetime genetic risk of developing glaucoma is 4%. This means that 4 out of 100 people with an average polygenic risk will develop glaucoma.

2. What does my test result mean to me?

- Your result suggests you have a similar risk of developing glaucoma compared to other people with an average genetic risk.
- Your result does not mean that you have glaucoma now.
- Even if your result shows that you are at average risk, you could still develop glaucoma.
- This result does not change your current screening or management program. This means that you should continue to follow advice from your eye care provider if you have one.
- The current recommendations for the population are to have regular eye health checks from the age of 50 years.
- Please refer to the back of the page for more information on glaucoma.

3. What are the limitations of the test?

- These results have not been validated in an accredited testing laboratory. Therefore, they should not be used as part of your clinical management.
- PRS results represent a probability of individual disease risk and are therefore not diagnostic.
- This result may change with time as new versions of the PRS become available.
- This test only estimates your risk of primary-open angle glaucoma (the most common subtype of glaucoma). It does not estimate your risk of other types of glaucoma or other conditions.
- Although the polygenic score predicts risk in many ancestries, it has been best validated in individuals of European ancestry.
- This test does not account for all genetic changes known to cause glaucoma. Therefore, your risk may be higher, especially if you have a strong personal family history of glaucoma.
- Results should be interpreted in conjunction with other known clinical risk factors. It does not take into account non-genetic risk factors.

Polygenic Risk Score (PRS) Report - Glaucoma

Firstname: Surname: ID:

DOB: Date of reporting:

1. **PRS Result - Glaucoma**

Your result - high risk

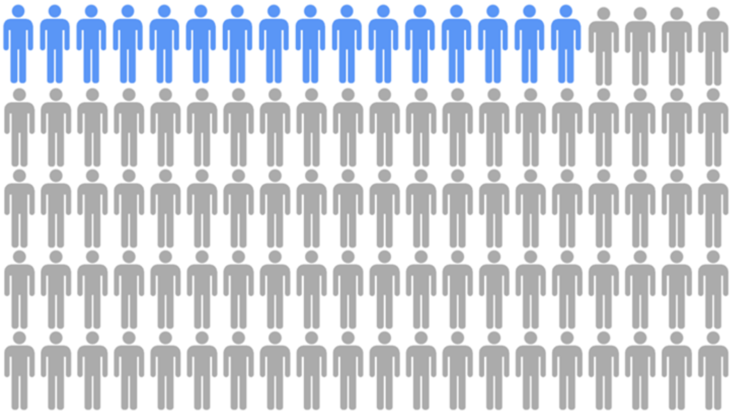

Your lifetime genetic risk of developing glaucoma is 16%. This means that 16 out of 100 people with the same polygenic risk result as you will develop glaucoma.

Average population

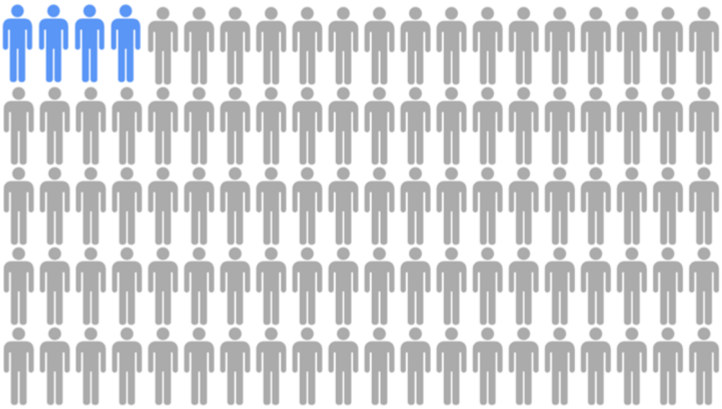

The average population lifetime genetic risk of developing glaucoma is 4%. This means that 4 out of 100 people with an average polygenic risk will develop glaucoma.

2. **What does my test result mean to me?**

- Your result suggests you are 4 times more likely to develop glaucoma compared to other people with an average genetic risk.
- Your result does not mean that you have glaucoma now.
- Even if your result shows that you are at high risk, it does not mean that you will definitely develop glaucoma.
- This result does not change your current screening or management program. This means that you should continue to follow advice from your eye care provider if you have one.
- The current recommendations for the population are to have regular eye health checks from the age of 50 years.
- Please refer to the back of the page for more information on glaucoma.

3. **What are the limitations of the test?**

- These results have not been validated in an accredited testing laboratory. Therefore, they should not be used as part of your clinical management.
- PRS results represent a probability of individual disease risk and are therefore not diagnostic.
- This result may change with time as new versions of the PRS become available.
- This test only estimates your risk of primary-open angle glaucoma (the most common subtype of glaucoma). It does not estimate your risk of other types of glaucoma or other conditions.
- Although the polygenic score predicts risk in many ancestries, it has been best validated in individuals of European ancestry.
- This test does not account for all genetic changes known to cause glaucoma. Therefore, your risk may be higher, especially if you have a strong personal family history of glaucoma.
- Results should be interpreted in conjunction with other known clinical risk factors. It does not take into account non-genetic risk factors.

Polygenic Risk Score Report - Glaucoma

Polygenic Risk in Detail

- Researchers have identified genetic variants which are associated with glaucoma by comparing those with the disease to those without.
- Your test results were based on 2673 genetic changes (or variants) that we know influence a person's risk of developing glaucoma.
- Polygenic risk scores add up all of the genetic variants associated with a particular condition that a person has, accounting for how strongly they are associated with the condition.
- Polygenic risk scores give an estimation of a person's genetic risk for a particular condition compared to other people within the population.

For a full explanation on genetic risk and calculation of PRS:
https://www.genome.gov/Health/Genomics-and-Medicine/Polygenic-risk-scores

Frequently Asked Questions

What is glaucoma?

- Glaucoma is a group of neurodegenerative conditions that affect the optic nerve.
- Glaucoma is usually a complex disease, influenced by both genetic and environmental factors.
- Primary open-angle glaucoma is the most common form of glaucoma:
    - Open-angle means the area where the fluid drains out of the eye is not obstructed.
    - Primary means there is no other known cause (such as trauma or surgery).

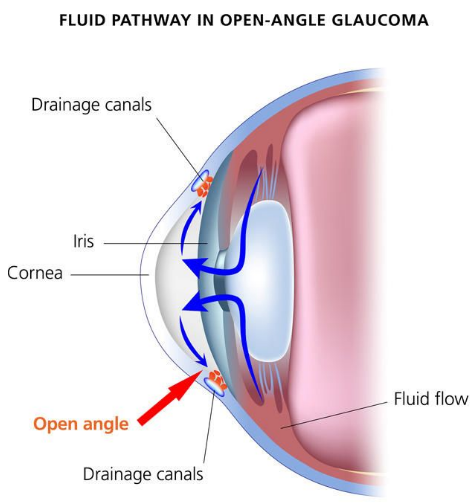

What are the symptoms?

- Usually there are no symptoms in early disease.
- Vision loss may only be noticeable in later stages of disease.
- Vision loss from glaucoma is irreversible and cannot be restored.
- Only an eye exam performed by an eye care provider can tell if someone has glaucoma.

Are there any risk factors?

- Yes, there are a number of risk factors (examples listed below):
- Family history of glaucoma:
    - Those who have a first-degree relative (parent, sibling, child) with glaucoma are at almost 10 times increased risk of also developing glaucoma compared to those who do not;
- African ancestry;
- Age over 50 years;
- Elevated eye pressure.

What are the treatment options?

- Treatment options are highly effective at slowing or preventing disease progression in most people.
- Treatments include topical eye drops, laser therapy, and in very advanced cases, surgery.

Resources - for more information

Glaucoma Australia:
https://glaucoma.org.au/home

Glaucoma Australia
Saving sight

Vision Australia:
https://www.vision2020australia.org.au/

VISION 2020 THE RIGHT TO SIGHT AUSTRALIA
