# Supplementary material for: Impact of polygeNic risk score for glaucoma on psycHosocial ouTcomes (INSiGHT) study protocol
Source: PLoS One. 2024 Dec 26;19(12):e0312390. doi: 10.1371/journal.pone.0312390 (PMC11670974; doi:10.1371/journal.pone.0312390)
Supplement: S2 File — (DOCX) [file pone.0312390.s002.docx]

**S2 File: Measures**

1. **Sociodemographic characteristics:**

What is your family cultural background (e.g. Australian, English, Chinese, etc…)?
*Free text response*

To which ethnic group/s do you most belong to?

Answers: Indigenous Australian / Torres Strait Islander / African / Asian / Caucasian / Hispanic / Middle Eastern / Pacific Islander / Unknown / Other (please specify)

What is the highest level of education you have completed?

Answers: University / Vocational/Technical certification / High school certification / No education completed

Are you currently employed, or performing paid work?

Answers: Employed / Unemployed / Retired / Other

Do you have biological children?

Answers: Yes / No

What language do you speak most at home?
*Free text response*

1. **Eye screening behaviour:**

Excluding the eye exam you may have had as part of this research project, when was the last time you had your eyes tested?

Answers: More than 2 years / Within 1-2 years / Within 6-12 months / Within 6 months / Never

How often do you have your eyes tested by an optometrist or an ophthalmologist?

Answers: More than every 2 years / Every 2 years / Annually / 6 monthly / 3 monthly / Never

What is the main reason for having your eyes tested?

Answers: Glaucoma check / Other existing eye condition / General vision-related check / Other (please explain)

How likely is it that you will have an eye test within the next 12 months?

Answers: Very Likely / Likely / Somewhat Likely / Unsure / Somewhat Unlikely / Unlikely / Very Unlikely

1. **Glaucoma status:**

Were you informed that you have borderline or high intraocular pressure?
Answers: Yes / No / Unsure

Have you been diagnosed with glaucoma?
Answers: Yes / No / Unsure

Does anyone in your family have glaucoma?

Answers: Yes / No / Unsure

If yes, how many? *Free text response*

How are they related to you? *Free text response*

1. **Health belief model measures:**
   1. Perceived glaucoma severity:

How serious would it be if you were to lose vision from glaucoma someday?

Answers: Not serious at all / Not serious / Somewhat not serious / Somewhat serious / Serious / Extremely serious

- 1. Perceived glaucoma risk:

What do you think are your chances of developing glaucoma in the future on a scale of 0-100% where: 0% = no chance of ever developing glaucoma, 100% = will definitely develop glaucoma someday.

*Scale*

What do you think are your chances of developing glaucoma sometime in the future compared to?

- An average person the same age and gender as you?
- An average person the same age and gender as you and with a similar family history of glaucoma as you?

Answers: Much Higher / Higher / Same / Lower / Much Lower

- 1. Response efficacy (genetic results):

Below is a list of factors other people have given for their decision to access their genetic information. Please indicate the extent to which you agree or disagree with each of the statements below.

If an option is not applicable to you, select the option that makes the most sense to you.

I am confident that receiving my genetic results will allow me:

1. To be able to take appropriate measures regarding my glaucoma risk and future eyesight
2. To be more certain about my glaucoma risk
3. To prepare for the future
4. To confirm my belief that I have a genetic risk of developing glaucoma
5. To be able to provide advice to my children about their potential risk
6. To be able to provide advice to my family members about their potential risk
7. To help research

Answers: Strongly Agree / Agree / Somewhat Agree / Somewhat Disagree / Disagree / Strongly Disagree

Are there any other factors that may influence (or have influenced) your decision about receiving your genetic results? Please specify the factor(s) and how it has influenced your decision.
*Free text response*

- 1. Response efficacy (eye test)

Below is a list of factors other people have given for their decision to have an eye test. Please indicate the extent to which you agree or disagree with each of the statements below.

If an option is not applicable to you, select the option that makes the most sense to you.

I am confident that an eye test (with an optometrist) will:

1. Detect glaucoma
2. Help me take appropriate measures regarding glaucoma
3. Help me prepare for the future
4. Help me provide advice to my family members about their potential risk
5. I am confident that glaucoma can be treated
6. I am confident that loss of vision from glaucoma can be prevented
7. I am confident that loss of vision from glaucoma can be minimised

Answers: Strongly Agree / Agree / Somewhat Agree / Somewhat Disagree / Disagree / Strongly Disagree

- 1. Response cost (genetic results):

Below is a list of factors other people have given for their decision not to access their genetic information. Please indicate the extent to which you agree or disagree with each of the statements below.

1. I am concerned about coping with the information emotionally
2. I am concerned about whether there is ongoing support upon receiving genetic results
3. I am concerned about the accuracy of the genetic results
4. I am concerned about the impact of the genetic information on my family members
5. I am concerned about how my genetic information will be used
6. I am concerned whether it could affect health insurance
7. I am concerned about the cost of a genetic test if it becomes part of standard clinical care
8. I am concerned about attending ongoing follow-up appointments
9. I am concerned it conflicts with my religious, cultural and/or spiritual beliefs
10. I am concerned there is insufficient knowledge among my healthcare providers on genetic testing and risk for glaucoma
11. I do not have a very good understanding of how my genetic results will inform me about my glaucoma risk
12. I do not believe that my genetic results will provide me with information regarding my glaucoma risk

Answers: Strongly Agree / Agree / Somewhat Agree / Somewhat Disagree / Disagree / Strongly Disagree

Are there any other factors that have influenced your decision about not receiving your genetic results? Please specify the factor(s) and how it has influenced your decision.
*Free text response.*

- 1. Response cost (eye test):

Below is a list of reasons other people have given for their decision not to have an eye test. Please indicate the extent to which you agree or disagree with each of the statements below.

If an option is not applicable to you, select the option that makes the most sense to you.

1. I do not know where an eye test is available to me
2. I will find it difficult to travel to my eye test
3. I understand why it is important to schedule an eye test
4. I believe that glaucoma will cause me to lose vision
5. I have too much stress in my life
6. I have a really hard time remembering to consider my eye health
7. I believe that if I am diagnosed with glaucoma, I will incur additional future costs
8. I have to go to other appointments regarding my health and/or other competing commitments
9. I find eye tests uncomfortable and/or painful

Answers: Strongly Agree / Agree / Somewhat Agree / Somewhat Disagree / Disagree / Strongly Disagree

- 1. Self-efficacy (Genetic results):

This section looks at your thoughts and feelings about receiving your polygenic risk score for glaucoma. Please indicate the extent to which you agree or disagree with each of the statements below.

If an option is not applicable to you, select the option that makes the most sense to you.

I am confident I would choose to receive my result even if:

1. My family does not want me to
2. The results cannot be explained by current scientific knowledge about genes and disease
3. The results could uncover unwanted information about my disease risk
4. I had to attend genetic counselling before and after receiving my results
5. I had to pay for an appointment
6. I had to communicate the results to my family
7. I had to communicate the results to insurance providers/future employer

Answers: Strongly Agree / Agree / Somewhat Agree / Somewhat Disagree / Disagree / Strongly Disagree

- 1. Self-efficacy (Eye test):

This section looks at your thoughts and feelings about having an eye test. Please indicate the extent to which you agree or disagree with each of the statements below.

If an option is not applicable to you, select the option that makes the most sense to you.

I am confident I will schedule and attend an eye test even if:

1. There is no eye test clinic close to my residence
2. I will have to travel far to access a clinic
3. My family does not want me to
4. I could uncover unwanted information about my eye health
5. I had to pay for an appointment
6. There is no one to remind me
7. I am busy
8. I worry about future commitments
9. I have no symptoms of abnormal eye health
10. I feel unwell
11. I have been told I have a relatively low risk of developing glaucoma

Answers: Strongly Agree / Agree / Somewhat Agree / Somewhat Disagree / Disagree / Strongly Disagree

- 1. Cues to action:

This section looks at ways that may prompt you to have an eye test. Please indicate the extent to which you agree or disagree with each of the statements below.

If an option is not applicable to you, select the option that makes the most sense to you.

1. My family encourages me to schedule an eye test
2. My friend/s encourages me to schedule an eye test
3. My doctor encourages me to schedule an eye test
4. I schedule reminders in my calendar notifying me of my eye test date/s
5. I receive reminders from my optometrist or eye health clinic notifying me on when my eye tests are due

Answers: Strongly Agree / Agree / Somewhat Agree / Somewhat Disagree / Disagree / Strongly Disagree

1. **Uncertainty avoidance:**

Below is a list of comments made by people when deciding whether to receive their genetic results. Please indicate how much you agree or disagree with each statement by selecting the answer that best represents your views.

1. I would rather receive my genetic results, and be more certain about my future health, even if the result is bad news
2. I would like to know now how likely I am to develop glaucoma so I can get used to the news
3. If I didn’t receive my genetic results I would always be wondering whether I was at risk to develop glaucoma
4. The relief I would get from a good result makes it worth the risk that the result is bad
5. I think it is tempting fate to ask questions about future illness
6. I would rather not know my results, than find out I am at risk to develop glaucoma
7. Knowing my genetic results would mean I felt more in control
8. It is better to know that I am at risk to develop glaucoma, even if I can’t prevent it

Answers: Strongly Agree / Agree / Neither Agree or Disagree / Disagree / Strongly Disagree

1. **Glaucoma anxiety:**

Below is a list of comments made by people during various life events. Please tick the box corresponding to the statement that indicates how frequently each comment was true for you in the past week regarding your chances of developing glaucoma. If any of these responses did not occur, tick the “not at all” box.

1. I thought about it when I didn't mean to
2. I avoided letting myself get upset when I thought about it or was reminded of it
3. I tried to remove it from my memory
4. I had trouble falling asleep or staying asleep because of pictures or thoughts about it that came into my mind
5. I had waves of strong feelings about it
6. I had dreams about it
7. I stayed away from reminders of it
8. I felt as if it hadn’t happened or wasn't real
9. I tried not to talk about it
10. Pictures about it popped into my mind
11. Other things kept making me think about it
12. I was aware that I still had a lot of feelings about it, but I didn't deal with them
13. I tried not to think about it
14. Any reminder brought back feelings about it
15. My feelings about it were kind of numb

Answers: Often / Sometimes / Rarely / Not at all

1. **Knowledge of glaucoma:**

In this section we would like to find out what you know about glaucoma risk and detection.

For each of the following statements please indicate if you believe it to be True, False or Don’t Know. If you are unsure or don’t know the answer, please tick the “don’t know” box.

1. All individuals at high risk for glaucoma will develop glaucoma
2. The interpretation of a high or low risk is the same for everyone
3. Common risk variants associated with glaucoma risk also increases a person’s risk for other eye diseases
4. There is more than one DNA change that can increase a person’s risk for glaucoma
5. It is possible to be diagnosed with glaucoma solely due to chance
6. Most individuals who develop glaucoma do not have family history of the disease
7. A person inherits DNA changes associated with glaucoma risk from both parents
8. A person may be at increased risk for glaucoma if they have several close relatives with glaucoma
9. If a person has a high risk for glaucoma there are screening and preventative options available
10. An eye test can only detect glaucoma when it is more advanced
11. Early detection of glaucoma means a greater chance of not losing vision
12. A person with early glaucoma may not be aware that they have the disease

Answer option for participants: True / False / Don’t know

1. **Genetic determinism:**

Please indicate the extent to which you agree or disagree with each of the statements below:

1. A person with a genetic risk to glaucoma will eventually develop glaucoma
2. The environment has far greater significance to the development of glaucoma than a person’s genes
3. Developing glaucoma is a natural result of having a genetic predisposition to glaucoma
4. People who have glaucoma are likely to have a genetic predisposition to glaucoma

Answers: Strongly Agree / Agree / Somewhat Agree / Somewhat Disagree / Disagree / Strongly Disagree

1. **Stressful life events:**

Below is a list of various stressful life events that people can experience. Please tick the box below, either ‘Yes’ or ‘No’ to indicate if any of the events below occurred for you in the past year.

1. You yourself suffered a serious illness, injury or an assault
2. A serious illness, injury or assault happened to a close relative
3. Your parent, child or spouse died
4. A close family friend or another relative (aunt, cousin, grandparent) died
5. You had a separation due to marital difficulties
6. You broke off a steady relationship
7. You had a serious problem with a close friend, neighbour or relative
8. You became unemployed or you were seeking work unsuccessfully for more than one month
9. You were sacked from your job
10. You had a major financial crisis
11. You had problems with the police and a court appearance
12. You moved to a new house
13. Something you valued was lost or stolen

Answers: Yes / No / Prefer not to say

1. **Generalised anxiety, stress and depression:**

Please read each statement and select the answer that indicates how much the statement applied to you over the past week. There are no right or wrong answers.

1. I found it hard to wind down
2. I was aware of dryness of my mouth
3. I couldn't seem to experience any positive feeling at all
4. I experienced breathing difficulty (eg, excessively rapid breathing, breathlessness in the absence of physical exertion)
5. I found it difficult to work up the initiative to do things
6. I tended to over-react to situations
7. I experienced trembling (eg, in the hands)
8. I felt that I was using a lot of nervous energy
9. I was worried about situations in which I might panic and make a fool of myself
10. I felt that I had nothing to look forward to
11. I found myself getting agitated
12. I found it difficult to relax
13. I felt down-hearted and blue
14. I was intolerant of anything that kept me from getting on with what I was doing
15. I felt I was close to panic
16. I was unable to become enthusiastic about anything
17. I felt I wasn't worth much as a person
18. I felt that I was rather touchy
19. I was aware of the action of my heart in the absence of physical exertion (eg, sense of heart rate increase, heart missing a beat)
20. I felt scared without any good reason
21. I felt that life was meaningless

Answers: Applied to me very much, or most of the time / Applied to me to a considerable degree, or a good part of time / )/ Applied to me to some degree, or some of the time / Did not apply to me at all

1. **Test-related distress:**

The following questions ask about how you felt after receiving your genetic test results. Please indicate how much you had each specific feeling in the past 2 weeks by circling the one answer for each question:

1. How upset did you feel about your genetic test result?
2. How happy did you feel about your genetic test result?
3. How anxious or nervous did you feel about your genetic test result?
4. How relieved did you feel about your genetic test result?
5. How sad did you feel about your genetic test result?
6. How frustrated did you feel that there are no definite disease prevention guidelines for you?
7. How uncertain did you feel about what your genetic test result means for you?
8. How uncertain did you feel about what your genetic test result means for your child(ren) and/or family’s risk of disease?
9. How much did you feel that you understood clearly your choices for disease prevention or early detection?
10. How concerned did you feel that your genetic test result would affect your health insurance status?
11. How helpful was the information you received from your genetic test result in planning for the future?
12. How concerned did you feel that your genetic test result would affect your employment status?

Answers: A great deal / A good deal / Somewhat / A little / Not at all

1. **Recall and interpretation of results:**

What was your risk of developing glaucoma based on your polygenic risk score?

*Free text response*

What was your genetic result associated with glaucoma risk?

Answers: It is higher than the general population / It is lower than the general population / It is the same as the general population / I do not remember/I am unsure of my genetic result / Other (please specify)

1. **Result expectation:**

If you have a personal history of glaucoma, please answer this question. Otherwise, please jump to the next question.

- - In line with your personal history, did you anticipate getting this test result?
  - According to your family history of glaucoma, did you anticipate getting this test result?

Answers: Yes, my result fits with my personal history of glaucoma / No, my result does not fit with my personal history of glaucoma / Unsure, I was not sure what to expect based on my personal history of glaucoma / I don’t believe there is a relationship between my personal history and this test result / Don’t know (please specify)

1. **Decisional regret:**

Please reflect on your decision to receive your polygenic risk score for glaucoma. Please respond to each of the following statements by selecting the answer that best reflects how you feel:

1. It was the right decision
2. I regret the choice that was made (reverse)
3. I would make the same choice if I had to do it over again
4. The choice did me a lot of harm (reverse)
5. The decision was a wise one

Answers: Strongly Agree / Agree / Neither Agree or Disagree / Disagree / Strongly Disagree

1. **Eye screening follow up:**

Have you had your eyes tested since you received your genetic results?

Answers: Yes / No

What was the main reason for having your eyes tested?

Answers: Glaucoma check / Other existing eye condition / General vision-related check / Other (please explain)

Have you been informed that you have borderline or high intraocular pressure since receiving your genetic results?

Answers: Yes / No / Unsure

Have you been diagnosed with glaucoma since receiving your genetic results?

Answers: Yes / No / Unsure
